# Hindmilk as a Rescue Therapy in Very Preterm Infants with Suboptimal Growth Velocity

**DOI:** 10.3390/nu15040929

**Published:** 2023-02-13

**Authors:** Belal N. Alshaikh, Jannette Festival, Adriana Reyes Loredo, Kamran Yusuf, Zainab Towage, Tanis R. Fenton, Christel Wood

**Affiliations:** 1 Neonatal Gastroenterology and Nutrition Program, Department of Pediatrics, Cumming School of Medicine, University of Calgary, Calgary, AB T2N2T9, Canada; 2 Community Health Sciences, Institute of Public Health, Cumming School of Medicine, University of Calgary, Calgary, AB T2N2T9, Canada; 3 Alberta Children’s Hospital Research Institute, University of Calgary, Calgary, AB T2N2T9, Canada; 4 NorthernStar Mothers Milk Bank, Calgary, AB T2H2A7, Canada; 5 Department of Pediatrics, Cumming School of Medicine, University of Calgary, Calgary, AB T2N2T9, Canada; 6 Nutrition Services, Alberta Health Services, Calgary, AB T2N2T9, Canada; 7 Alberta Health Services, Calgary, AB T2N2T9, Canada

**Keywords:** extrauterine growth restriction, growth faltering, postnatal growth failure

## Abstract

Despite advances in neonatal nutrition, very preterm infants remain at increased risk of extrauterine growth faltering. This prospective study aimed to examine the effect of hindmilk, the milk at the end of a breast expression session, on growth and plasma fatty acids (FAs) of infants born <30 weeks’ gestation who had been on full enteral feeds for ≥2 weeks and had a weight gain of <15 g/kg/day despite optimizing energy and protein intakes. Weight and plasma FAs were assessed before and two weeks after feeding hindmilk. Growth anthropometrics were assessed weekly for four weeks. Paired t-tests and multiple linear regression were used for statistical analyses of data from 34 infants and their 29 mothers. There was a significant increase in weight gain in the two weeks after feeding hindmilk (MD 3.9, 95%CI 1.2–6.5 g/kg/day). Weight Z-scores were larger at two weeks (MD 0.61, 95%CI 0.02–1.20) and onwards. Head circumference Z-scores were larger at three weeks (MD 0.83, 95%CI 0.20–1.47) and onwards. Plasma linoleic acid (LA) and α-linolenic acid (ALA) increased after feeding hindmilk. In conclusion, hindmilk may improve weight and head growth and increase LA and ALA in very preterm infants with suboptimal growth. A large randomized controlled trial is required to examine and validate the potential benefits of hindmilk.

## 1. Introduction

Despite advances in neonatal nutrition, very preterm infants remain at increased risk of extrauterine growth faltering [1,2]. Poor growth in preterm infants is associated with suboptimal neurodevelopmental outcomes [3,4]. Although multifactorial, infants’ growth faltering commonly results from deficits in energy and protein intakes. These deficits occur despite utilizing human milk multi-nutrient fortifiers (HMFs) as a standard of care in neonatal intensive care units (NICUs).

Fat constitutes the main energy source in human milk and accounts for 40–50% of caloric intake [5]. Hindmilk, the milk at the end of a breast expression session, has higher fat and energy content compared with foremilk. Fat concentration in hindmilk is 2–3 times higher than in foremilk, and approximately 1.3–1.6 times higher than in composite milk, milk obtained over the entire course of an expression session [6,7,8]. Hindmilk has the advantage of providing higher energy intake without the need to increase the volume or osmolarity of the milk consumed. Therefore, it may be useful for very preterm infants whose enteral feed volumes are limited by the immaturity of their gastrointestinal system or the need to restrict fluid intake due to evolving bronchopulmonary dysplasia (BPD). 

Breastmilk is also the main source of essential fatty acids (EFAs) in the postnatal period. EFAs are essential for brain development, visual functions, and growth [9]. Feeding hindmilk presents theoretical advantages of providing higher EFAs intakes for preterm infants. Thus, hindmilk could be favorable to the optimal development and growth of preterm infants. 

The primary aim of this study was to assess the effect of feeding hindmilk on the growth velocity of very preterm infants with suboptimal growth velocity. The secondary aim was to examine the impact of feeding hindmilk on infants’ plasma fatty acid profile. 

## 2. Materials and Methods

### 2.1. Patients and Study Design

This was a prospective cohort study conducted between January 2019 and June 2022 in the 39-bed level III NICU at Foothills Medical Center in Calgary, Alberta, Canada. The study was approved by the Conjoint Research Ethics Board of the University of Calgary (REB18-0195, date: 2 April 2018). Written informed consent was obtained from mothers before enrollment. 

Infants born <30 completed weeks gestation and weighing <1500 g to mothers with enough milk supply (≥150% of infant’s daily needs) were on full enteral feeds for more than 2 weeks and that had slow weight gain (<15 g/kg/day, calculated using the average weight as the denominator), despite optimization of energy and protein intakes, were eligible. Optimization of nutritional intakes was achieved by our two neonatal registered dietitians to meet recommended daily intakes of energy intake (110−135 Kcal/kg/day) and protein (4.0−4.5 g/kg/day for infants weighing <1000 g and 3.5−4.2 g/kg/day for infants weighing 1000−1500 g) [10]. Infants were excluded if they were small for gestational age (SGA) or had major congenital or chromosomal anomalies. Full enteral feeding was defined as a minimum of 140 mL/kg/day that was sustained for 3 days with no parenteral nutrition. 

The primary outcome of the study was the rate of weight gain in the two weeks after feeding hindmilk. Weight gain (g/kg/day) was calculated using the average 2-point method [(W2 − W1)/([(W2 + W1)/2]/1000) /number of days] (W = weight expressed in grams) [11]. The secondary outcomes were a) changes in anthropometric (weight, head circumference, and length) Z-scores after 4 weeks, and b) changes in fatty acids profile 2 weeks after feeding hindmilk. Plasma fatty acids were analyzed by OmegaQuant Analytics, Sioux Falls, SD, USA (Supplementary Materials) [12,13,14,15]. 

### 2.2. Enteral Nutrition 

Mother’s own milk (MOM) was the exclusive source of enteral nutrition for study subjects. None of the subjects required pasteurized donor human milk in the 2 weeks prior to hindmilk fractioning or during the study. Weight-based enteral feeding tables were used as per our NICU guidelines, and daily feeding goals were posted at each infant’s bedside. Nutrient fortification commenced at 80 mL/kg/day, and parenteral nutrition was stopped once feeding volume reached 100–120 mL/kg per day and tolerance was observed. Fortification began at 1 package/50 mL human milk, and when the infant reached 100 mL/kg/day it was increased to 2 packages/50 mL human milk (approximately 0.78 kcal/mL). Similac Human Milk Fortifier Extensively Hydrolyzed Protein Concentrated Liquid (Abbott Nutrition, Tipp City, OH, USA) was used for nutrient enrichment. In infants with protein intake less than recommended values, Similac Extensively Hydrolyzed Protein Concentered Liquid (Abbott Nutrition, Tipp City, OH, USA) was prescribed at 0.5–1.5 g/kg/day amounts after nutrient fortification had reached 0.78 kcal/mL. Enteral feeds were further concentrated if weekly weight gain was <15 g/kg/day. Powdered hydrolyzed-protein formula (Nutramigen, Mead Johnson, Evansville, OH, USA) was prescribed to further concentrate feeds to approximately 0.92 kcal/mL (27 kcal/oz) as needed. Infants identified to have suboptimal growth velocity were followed daily by registered dietitians.

Probiotics were prescribed in infants weighing ≥1000 g as per our NICU protocol. Standardized multivitamin drops were provided daily, containing 375 IU of vitamin A, 200 IU of vitamin D, and 17.5 mg of vitamin C, with an additional 400 IU of vitamin D until the infant reached 37 weeks corrected gestational age. Iron supplement was prescribed at 2–3 mg elemental iron/kg/day beginning at 14 days of life. 

All infants were weighed daily on electronic scales with a precision of 10 grams by nursing personnel, using the In-Bed scale (GiraffeTM OmniBedTM, Ohmeda Medical, Orang City, FL, USA). Other anthropometric measurements, length, and head circumference were taken by nursing personnel on admission and then weekly till discharge with a newborn length board (Length BoardsTM, Ellard Instrumentation Ltd., Monroe, MI, USA) and measuring tape, respectively. Infants were classified as being small for gestational age at admission to the NICU if they were below the 10th centile using Fenton’s Growth Curves [16]. Levels of humidity and temperature inside the incubators were used as per our standardized thermoregulation protocol for very preterm infants. BPD was defined according to Child Health and Human Development as the requirement for positive pressure support (continuous positive airway pressure or high flow nasal cannula ≥1 liter per minute), or oxygen dependency at 36 corrected gestational age [17]. Retinopathy of prematurity (ROP) was defined according to the international classification1 or requiring treatment [18]. 

### 2.3. Milk Separation

Once the mother’s consent was obtained, mothers were counseled by the study lactation support consultant on techniques to separate their milk during routine pumping sessions. All mothers used the double-pumping system for milk expression (Medela, McHenry, IL, USA). Foremilk was defined as the milk collected for 3 min after the flow has begun [8]. Hindmilk was defined as the remainder of the subsequent milk fraction collected until complete breast emptying. Mothers labeled their milk as foremilk and hindmilk. Mothers froze foremilk for possible use later. Mothers continued to separate their milk during each pumping session until either their milk supply had decreased, their infants were breastfeeding on demand, or they reached 37 weeks corrected gestational age. 

Two fresh milk samples, 6 mL each, were collected once for milk content analysis at the start of the study. The first sample was from composite milk and the second sample was from hindmilk. Milk samples were analyzed using a near-infrared (NIR) instrument (Unity SpectraStar XL, Unity Scientific, Blaxland, Australia). Milk samples for fatty acid profiles and dried spots (filter papers, 30–100 µL) were collected at the same time. Blood samples for plasma fatty acid profiles were collected on dried spots and on the same day as milk sampling; the second sample was collected after 2 weeks. Dried blood and milk spots were initially placed in a refrigerator for <12 h and then stored in a −80 °C freezer. The collection of blood samples was coordinated with other blood tests obtained for clinical purposes. 

Data on fluid volumes, feeds, macronutrient intakes, and any change in the nutrition plan were collected from electronic charts. Average weight gain was collected weekly for 2 weeks before and 2 weeks after starting hindmilk. The clinical team was encouraged to not change the nutrition plan for 2 weeks after starting hindmilk. Daily weight and weekly length and head circumference were collected from the electronic charts. Furthermore, weight, length, and head circumference were used to calculate Z scores using Fenton Z scores completed gestational week calculator [19]. 

### 2.4. Statistical Analyses

Our local data (unpublished) indicated that very preterm infants with suboptimal growth velocity had an average weight gain of 13 g/kg/day (standard deviation (SD) = 7). A previous small study of 15 preterm infants showed a 7 g (SD = 4.4) increase in weight gain when hindmilk was fed [7]. However, our preliminary data from a quality improvement initiative to utilize hindmilk indicated a 4 g/kg/day increase in weight gain with hindmilk. The 4 g/kg/day increase was considered clinically significant given that it can be enough to restore appropriate weight gain and allow catch-up growth. Thus, we calculated the sample size based on this difference. Assuming a 2-sided α value of 0.05 and a power of 0.90, a sample size of 33 infants was required to detect the difference in average weight gain between the 2 study groups using a calculation of repeated measures in STATA software (STATA 11.0, College Station, TX, USA). 

Descriptive statistics using mean, median, SD, and interquartile range (IQR) were used to describe the study population. A paired t test was used to compare average weight gain before and after hindmilk use. Linear regression analysis was used to assess the association between weight gain and milk content, and to adjust for potential confounding factors that included changes in measured milk protein, gestational age at birth, infant sex, exposure to low-dose postnatal dexamethasone to facilitate extubation (DART protocol) [20], and BPD as a surrogate for severity of lung disease. For all tests, a probability of *p* value < 0.05 was considered statistically significant. STATA software was used for all analyses.

## 3. Results

### 3.1. Study Population

**Figure 1:**
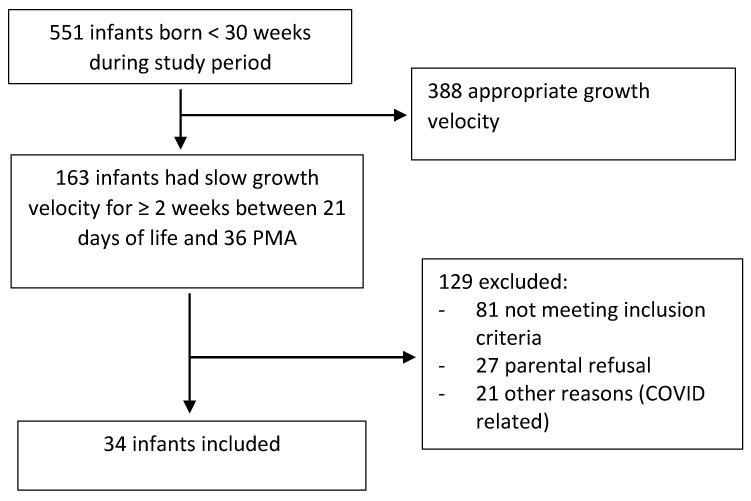
Study flow diagram.

A total of 551 infants were born <30 weeks gestation during the study period. One hundred sixty-three infants had slow growth velocity between 21 days of life and 35-week postmenstrual age (PMA). One hundred twenty-nine infants were excluded for; not meeting the inclusion criteria (*n* = 82), parental refusal to participate (*n* = 27), and other reasons (*n* = 21). Thirty-four infants completed the study (Figure 1). Maternal and neonatal baseline characteristics are summarized in Table 1. Thirty-three infants (97%) were on respiratory support at the time of enrollment; of these, two were on ventilation, and thirty were on non-invasive respiratory support. 

All mothers were able to provide hindmilk for two weeks. Four mothers were inconsistent in providing hindmilk between two and four weeks and stopped milk fractioning after four weeks due to decreased milk volumes. 

### 3.2. Enteral Nutrition and Milk Content

**Figure 2:**
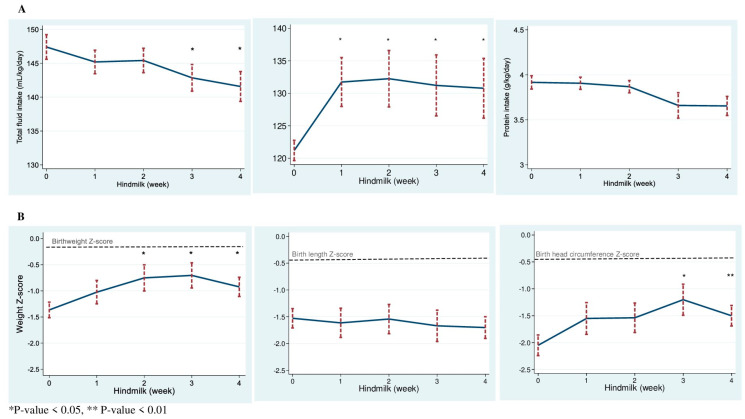
(**A**) Daily total fluid, energy, and protein intakes during the study period. (**B**) Z−scores at the start of hindmilk and after 4 weeks. dash bars are standard error of the mean.

Enteral feeding was initiated at a median age of 1 day (IQR 0, 1). The median age at starting hindmilk was 33.5 (IQR 26, 38.5) days. Total fluid intake slightly decreased in weeks 3 and 4 (Figure 2A). Total energy intakes were significantly higher after the introduction of hindmilk. It is important to note that we calculated the energy intakes based on the results of analyses for composite milk and hindmilk contents. (Figure 2A). The content of composite milk and hindmilk is summarized in Table 2. Compared with composite milk, hindmilk was more energy-dense and had a higher fat concentration. There were no significant differences in protein and carbohydrates between composite milk and hindmilk.

### 3.3. Growth and Anthropometric Measurements

**Figure 3:**
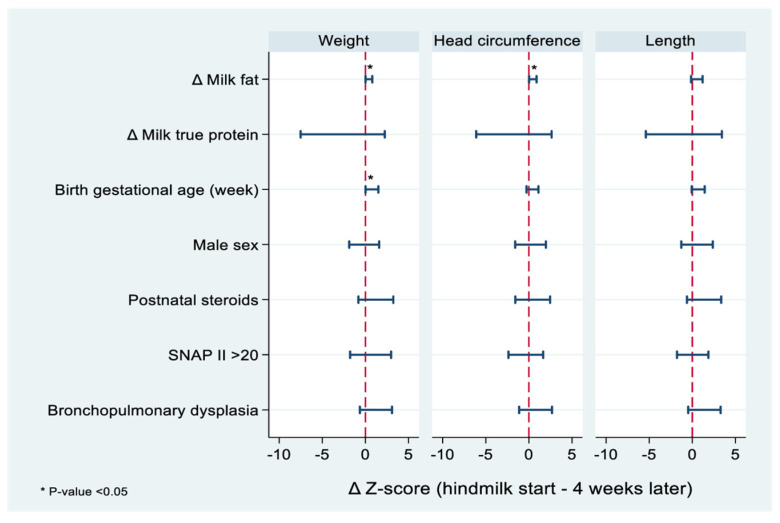
Multiple linear regression for associations between hindmilk fat content and anthropometric growth velocities in preterm infants. Δ Z−score is measured as a change in Z−score per every 1 g/dL difference in fat (Δ fat) and true protein (Δ true protein) between composite milk and hindmilk. The Δ fat and Δ true protein are based on a single analysis for composite milk and hindmilk.

There was a significant increase in the rate of weight gain in the 2 weeks after starting hindmilk compared with the prior 2 weeks (MD 3.9 g/kg/day, 95%CI 1.2, 6.5). Because the nutritional intakes and growth during the transitional period after reaching full enteral feeds were unstable in some infants, we compared weight gain between the latest week when the infants were on composite milk with the 2 weeks after hindmilk introduction. The detailed nutrient intakes during this period are summarized in Table 3. The mean difference in weight gain was 3.8 g/kg/day (95%CI 0.1, 7.5). Weight Z-scores were significantly larger at 2 (MD 0.61, 95%CI 0.02, 1.20), 3 (MD 0.62, 95%CI 0.05, 1.18), and 4 (MD 0.37, 95%CI 0.01, 0.73) weeks after starting hindmilk (Figure 3). Head circumference Z-scores were larger at 3 (MD 0.83, 95%CI 0.20, 1.47) and 4 (MD 0.52 (0.22, 0.81) weeks after starting hindmilk (Figure 3). Length Z-scores remained unchanged after starting hindmilk. 

Linear regression analysis showed a significant association between the difference in fat content (Δ fat) between composite milk and hindmilk, and weight Z-score catch-up (Δ) between the start of hindmilk and 4 weeks later (adjusted Δ Z-score 0.40, 95%CI 0.01, 0.8 for every 1 g increase in milk fat), Figure 3. There was similar association between Δ fat content, and head circumference Z-score catch-up (adjusted Δ Z-score 0.46, 95%CI 0.03, 0.88 for every 1 g increase in milk fat) for the 4 weeks duration. No association was found between Δ fat and changes in length Z-score catch-up (adjusted Δ Z-score 0.53, 95%CI −0.14, 1.19). All models were adjusted for milk protein, gestational age at birth, infant sex, exposure to low-dose postnatal dexamethasone to facilitate extubation, and BPD.

### 3.4. Biochemical Outcomes

**Figure 4:**
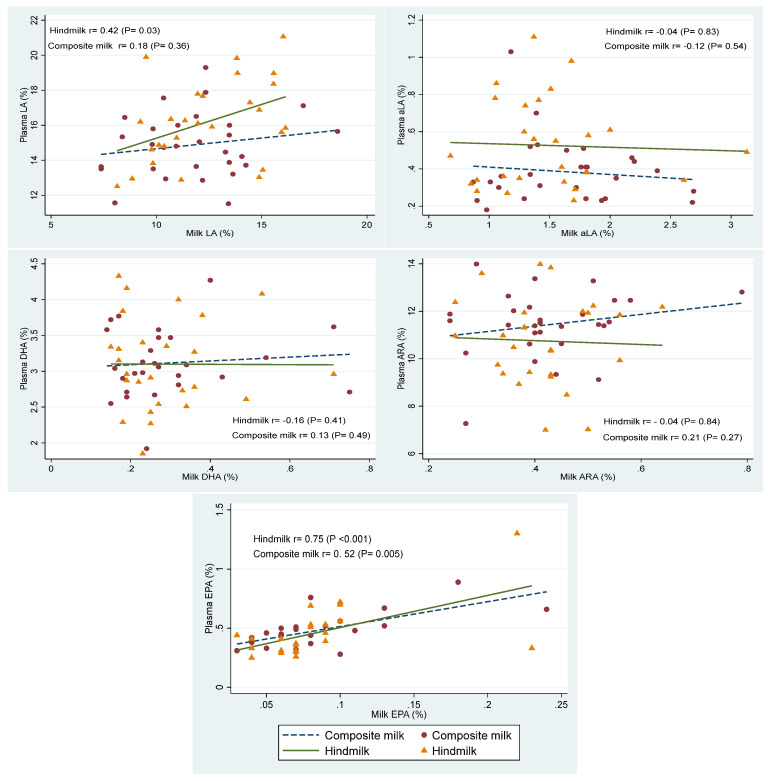
Correlations between milk and plasma bioactive fatty acids.

Oleic (C18:1 n9), palmitic (C16:0), and linoleic acid (LA; C18:2 n6) were the dominant fatty acids in milk (Table 4) and plasma (Table 5). Although all fatty acids concentrations were higher in hindmilk, only LA reached statistical significance (MD 16.2, 95%CI 3.7, 28.7 µg/mL). Changes in plasma fatty acids concentrations (%) are presented in Table 4. Plasma palmitoleic acid (C16:1 n7) decreased significantly after 2 weeks of hindmilk. Plasma LA and α-linolenic acid (ALA, C18:3 n3) increased by 1.1-fold and 1.3-fold, respectively, after 2 weeks of hindmilk. There were no significant differences in plasma arachidonic acid (ARA, C18:2 n6) or docosahexaenoic acid (DHA, C22:6 n3). Plasma LA had significant correlation with hindmilk LA (Figure 4).

## 4. Discussion

Our study indicates that feeding hindmilk to very preterm infants with growth faltering may result in larger weight gain and higher weight and head circumference Z-scores after four weeks. Concentrations of fat and energy were significantly higher in hindmilk and were associated with improved weight and head size growth. Feeding hindmilk resulted in higher plasma EFAs, LA, and ALA. There was no difference in milk fatty acids distribution between hindmilk and composite milk. 

Although the pathogenesis of suboptimal extrauterine growth is multifactorial, variations in energy intakes could explain approximately 45% of the growth deficits in preterm infants [21]. Meeting recommended dietary intakes (RDIs) in infants with suboptimal growth is often challenging due to interruption of feeding plans and important inter- and intraindividual variation in breastmilk protein, sodium, and fat content [22]. Our study included infants of mothers with breastmilk volumes that exceeded 1.5 times the infants’ needs. Current evidence suggests that breastmilk is likely to be diluted and has less energy and protein when expressed volumes exceed 400–450 mL/day [23,24]. Hindmilk provided additional fat and energy to support growth, while protein intake was already optimized in the 1–2 weeks prior to breastmilk fractioning. 

Fractionating breastmilk in our study resulted in approximately 1.4- and 1.16-fold increases in fat and energy of hindmilk content, respectively, compared with composite milk. This is in line with previous studies on the hindmilk of mothers of preterm infants [7,8]. Previous studies reported 1.2- and 1.1-fold increases in milk fat and energy concentrations, respectively, when hindmilk was compared with composite milk [7,8]. Thus, hindmilk offered a natural source of additional fat that potentially limited the postnatal energy deficits in our study participants. 

Our finding of increased weight gain is consistent with that in a previous study that used similar pre- and post-study design [7]. Valentine et al. reported a 7.0 (SD 4.4) g/kg/day increase in weight gain after using fortified hindmilk in 15 low-birth-weight infants [7]. The study examined weight gain for one week only. They found a significant increase in energy content and fat concentration of hindmilk compared with composite breastmilk with a significant correlation between weight gain and milk fat concentration. Similarly, Slusher et al. reported a mean weight gain of 18.8 g/day in 16 preterm infants weighing less than 1800 g at birth and fed exclusively unfortified expressed hindmilk in a Nigerian neonatal unit [25]. However, the study did not include a control group, and postnatal growth was compared with a proposed intrauterine growth velocity of 15 g/day without specifying average gestational age. In contrast, a randomized controlled trial of hindmilk feeding involving 20 preterm infants indicated no significant difference in weight, length, or head circumference between the experimental fortified hindmilk group and the control fortified composite milk group [26]. The authors collected hindmilk after only one minute of breastmilk expression, and the composition of fortified hindmilk and fortified composite milk was similar in terms of energy content and concentrations of fat and energy [26]. 

It is worth mentioning that the effect of hindmilk in our study was temporary. The discharge weight z-score returned to values that were presented at the time of hindmilk introduction. The transitional period between 33–34 weeks corrected gestational age, which matches the endpoints in our study, and hospital discharge is complex in extremely preterm infants on respiratory support. Healthcare providers typically try weaning infants off nasogastric tubes and non-invasive respiratory support during this period. In addition, the type of fortification and other nutritional support are usually modified to facilitate hospital discharge. Some infants in our cohort required PN after the study endpoints due to morbidities including late-onset sepsis, NEC, and the need for surgical ligation to close the patent ductus arteriosus. Furthermore, most of the preterm infants in our cohort were transferred to level II NICUs before discharge. Unfortunately, we were not able to track nutritional intakes after the transfer. 

Using a mathematical approach, a 1.3 g fat/dL difference between analyzed composite milk and hindmilk results in an actual nutritional difference of 1.07 g of fat (83% of milk volume in 100 mL of fortified HM) and 9.6 Kcal/dL, after considering the fortification with liquid HMF and assuming relatively equal fluid intakes between pre- and post-hindmilk. Because the fat absorption in preterm infants is significantly lower than the fat absorption rate used to define metabolizable energy of the Atwater factor (70–80% in preterm infants versus 96% in adults) [27], the metabolizable benefit of hindmilk could be estimated at about 0.8 g of fat or 7.2 kcal/100 mL corresponding to 1.16 g of fat and 10.4 kcal/kg/day, considering a volume intake of 145 ml/kg/day during the first two weeks of hindmilk. This energy difference does not explain all the observed weight gain differences. Metabolic and energy balance studies have indicated that the energy cost of growth is 4.5 (range 2.9–6.0) kcal/g of weight gain [28]. Thus, the contribution of hindmilk (10.4 kcal/kg/day estimated energy difference) could explain a weight gain difference of 2.3 (range 1.7-3.6) g/kg/day. Other factors such as improved clinical condition, lower energy expenditure due to improved lung functions, and decreased inflammation may have contributed to the higher rate of weight gain after hindmilk. 

Hindmilk is an attractive source of non-protein energy for improving growth in preterm infants because of its high energy density, lower osmolality compared with other fortification strategies, and its role in providing higher concentrations of soluble vitamins [8]. However, before embarking on a wide use of hindmilk in preterm infants, it is important to examine whether macronutrients and micronutrients meet or exceed recommended nutrient intakes, particularly for fat and fat-soluble vitamins [8]. There are indications that hindmilk may contain more fat-soluble vitamins than composite milk, and fortification may provide excessive amounts of fat-soluble vitamins to preterm infants born at less than 28 weeks gestation. The analyzed samples in our study were fresh samples of composite milk and hindmilk. However, the infants were fed mothers’ fortified hindmilk. A more comprehensive and frequent analysis of the hindmilk that is actually fed to the babies would have improved our understanding of nutrient intakes. 

Studies of optimal nutrition in preterm infants have primarily focused on the need for adequate protein intake to improve linear growth [29,30]. Use of the single-nutrient protein supplement in our study provided an opportunity to provide sufficient protein intake of 3.5–4.0 g/kg/day, as current evidence supports [29,31]. Optimization of protein intake in the two weeks prior to starting hindmilk in our study did not improve weight gain. The larger weight gain after hindmilk may denote the need for more energy to utilize protein in infants with high energy demand, particularly infants on non-invasive ventilation. Because of limited endogenous energy stores in very preterm infants, the added protein may be catabolized to produce energy rather than utilized for tissue accretion, given their high energy demand. 

There was a significant improvement in head circumference Z-score three and four weeks after feeding hindmilk. Previous studies on hindmilk did not reveal a significant change in head growth after hindmilk; however, these studies monitored head circumference for shorter durations [7,25,26]. It is noteworthy that provision of high early parenteral fat intake of 2–3 g/kg/day starting soon after birth resulted in a significant increase in head circumference at 36 weeks PMA [32,33]. A meta-analysis of 4 RCTs indicated a 0.67 (95%CI 0.25, 1.09) cm increase in head size at term equivalent age after high parenteral fat intake [33]. Higher energy and fat intake in the first two weeks after birth was associated with a lower incidence of brain lesions and dysmaturation at term equivalent age in very preterm infants [34]. Although these studies used parenteral fat, they alluded to the key role of adequate fat intake for brain growth and development [33,34]. Nonetheless, the improvement of head z scores in our study was not expected and may have happened by chance due to the small sample size. Larger studies are needed to validate our results. 

Plasma LA and ALA in our study increased by 1.1 and 1.3-fold, respectively, with no change in plasma ARA and DHA. LA and ALA are precursors for *n*-6 and *n*-3 long-chain polyunsaturated fatty acids (LCPUFAs). Dietary intakes of LA and ALA, and the balance between them, have the potential to affect the LCPUFA status and impact neural functions and tissue adiposity in preterm infants [35,36]. Intervention studies in formula-fed infants concluded that increasing ALA supply improves infant DHA status [37]. This is in contrast to studies showing no change in DHA in adults where other food LCPUFAs sources were consumed [38]. It is important to note that studies in infants were conducted using formulas lacking pre-formed LCPUFAs, and the ALA doses were substantially above the current recommended range for ALA [37]. The contribution of ALA as a precursor to DHA is likely to be limited, since pre-formed DHA consistently raises plasma DHA levels regardless of ALA intakes [39]. The human milk fortifiers used in our NICU have significant concentrations of DHA and ARA with *n*-6: *n*-3 ratio of 4.5:1.0. The use of human milk fortifier in both phases of our study may partly explain the lack of difference in plasma DHA and ARA levels with hindmilk.

### Strength and Limitations

Our study included more infants than previous studies and followed growth velocity for a longer duration. The study is the first to report plasma fatty acid distribution after feeding hindmilk. Our study has several limitations to acknowledge. First, the study did not have a control group to compare growth anthropometrics and fatty acids, so it is not known how much of the infants’ growth improvements were due to the hindmilk intervention and how much might have occurred without the intervention. Randomization or having a control group for the infants with suboptimal growth velocity was not logistically feasible or ethical in our NICU settings, because hindmilk was part of standardized practice guidelines that aim to optimize postnatal growth. It is worth noting that the only change for the infants in our cohort was the hindmilk, and there were no other changes to their diet. Second, due to logistical challenges, we analyzed the samples of composite milk and hindmilk at the beginning of the study only and before fortification. A more frequent and comprehensive analysis is required in future studies. Third, we did not perform body composition or magnetic resonance imaging to identify if hindmilk alters the body’s fat mass, fat-free mass, or brain volume and connectivity. Fourth, we did not follow up regarding the long-term growth or neurodevelopmental outcomes. Long-term follow-up studies are needed to further understand the impact of feeding fortified hindmilk.

## 5. Conclusions

In conclusion, hindmilk produced by mothers of very preterm infants born <30 weeks’ gestation has higher concentrations of fat and energy compared with composite milk. Feeding very preterm infants with suboptimal growth velocity their mothers’ fortified hindmilk may improve weight and head growth trajectories and plasma concentrations of LA and ALA. Whether the infants’ accelerated weight and head growth and increasing plasma LA and ALA improve long-term neurodevelopment and brain growth parameters remains to be explored. Larger studies are required to examine the benefits of hindmilk and validate our results. 

## Figures and Tables

**Table 1:** Maternal and Neonatal characteristics of study participants.

Characteristics	*n* = 34
Gestational age, week, mean (SD)	26.5 (1.6)
Birthweight, grams, mean (SD)	855 (208)
Maternal age, year, mean (SD)	31 (4.8)
Prim gravida mother, *n* (%)	17 (50.0)
Parity, median (IQR)	0 (0, 1)
Gestation hypertension, *n* (%)	3 (8.8)
Cesarean section, *n* (%)	23 (67.6)
Twin pregnancy, *n* (%)	12 (54.5)
Chorioamnionitis, *n* (%)	3 (8.8)
Antenatal steroid, *n* (%)	31 (91.1)
SNAP II, median (IQR)	9 (0, 20)
Duration of mechanical ventilation, median (IQR)	10 (3, 23)
Respiratory support at start, *n* (%) - Ventilation - Non-invasive - No support	2 (5.9) 31 (91.2) 1 (2.9)
Postnatal steroid, *n* (%)	7 (25.9)
Bronchopulmonary dysplasia, *n* (%)	13 (38.2)
Severe intraventricular hemorrhage, *n* (%)	2 (5.9)
Patent ductus arteriosus required treatment, *n* (%)	20 (58.8)
Severe retinopathy of prematurity, *n* (%)	1 (2.9)
Culture-proven sepsis, *n* (%)	2 (6.0)
Necrotizing enterocolitis, *n* (%)	1 (2.9)
Gestational age at discharge, mean (SD)	40.1 (2.9)
Age at starting hindmilk, day, median (IQR)	33.5 (26, 38.5)
Duration of parenteral nutrition during total hospital stay, day, median (IQR)	17 (11, 25)
Duration of first course parenteral nutrition, day, median (IQR)	14 (9, 23)
Duration of central line, day, median (IQR)	10 (6, 17)
Birthweight Z-score, mean (SD)	−0.21 (0.93)
Birth length Z-score, mean (SD)	−0.43 (1.26)
Birth head circumference z-score, mean (SD)	−0.44 (0.93)
Discharge weight Z-score, mean (SD)	−1.34 (1.0)
Discharge length Z-score, mean (SD)	−2.49 (1.42)

**Table 2:** Macronutrient content of composite milk and hindmilk.

Content	Composite Milk	Hindmilk	Mean Difference (95%CI)	*p*-Value
Crude protein, g/dL, mean (SD)	1.5 (0.3)	1.5 (0.3)	0.03 (−0.05, 0.12)	0.44
True protein, g/dL, mean (SD)	1.1 (0.2)	1.1 (0.2)	0.01 (−0.05, 0.08)	0.71
Fat, g/dL, mean (SD)	3.4 (1.0)	4.7 (1.8)	1.3 (0.7, 2.0)	<0.001
Carbohydrate, g/dL, mean (SD)	7.9 (0.3)	7.9 (0.3)	0.09 (−0.03, 0.22)	0.13
Calorie, kcal/dL, mean (SD)	68 (10)	79 (17)	11.9 (5.5, 18.3)	<0.001

**Table 3:** Estimated and actual intakes in the week before and the two weeks after feeding fortified hindmilk.

	Composite Milk (7 Days before Hindmilk)			Hindmilk (14 Days from Start)		
	Estimated Values Based on Composite Milk Reference	Values Based on Milk Analysis	Additional Fortification	Estimated Values Based on Composite Milk Reference before Adjustment *	Values Based on Hindmilk Analysis	Additional Fortification
Energy intake, mean (SD), Kcal/kg/day	100.3 (6.4)	100.8 (15.2)	19.3 (6.8)	97.1 (4.8) **	114.9 (20.5)	22.3 (10.9)
Protein intake, mean (SD), g/kg/day	1.77 (0.24)	1.58 (0.29)	2.15 (0.40)	1.60 (0.08)	1.57 (0.27)	2.28 (0.39)

* These values are based on composite milk reference given the unavailability of standard reference for hindmilk. ** Energy values need to be multiplied by ~1.1–1.2 to estimate the ones in hindmilk.

**Table 4:** Concentrations of milk fatty acid in composite milk and hindmilk.

Common Name	Fatty Acid	Composite Milk Content (µg/mL)	Hindmilk Content (µg/mL)	Mean Difference (µg/mL)	*p* Value
Capric acid	C10:0	8.5 (4.2)	9.7 (3.6)	1.2 (−0.5, 2.9)	0.18
Lauric acid	C12:0	37.8 (19.2)	40.7 (16.3)	2.9 (−3.9, 9.8)	0.39
Myristic acid	C14:0	38.3 (18.3)	43.9 (21.5)	5.6 (−3.2, 14.4)	0.20
Palmitic acid	C16:0	122.0 (63.6)	149.4 (73.3)	27.4 (−3.9, 58.7)	0.06
Palmitelaisic acid	C16:1 n7t	0.6 (0.3)	0.7 (0.4)	0.1 (−0.1, 0.3)	0.20
Palmitoleic acid	C16:1 n7	12.1 (7.6)	13.7 (6.6)	1.6 (−1.4, 4.7)	0.28
Stearic acid	C18:0	31.8 (17.0)	38.1 (19.5)	6.2 (−2.0, 14.5)	0.13
Elaidic acid	C18:1t	3.1 (1.8)	3.7 (2.5)	0.6 (−0.4, 1.6)	0.21
Oleic acid	C18:1 n9	217.5 (85.6)	252.6 (101)	35.0 (−10.9, 81.0)	0.13
Linoelaidic acid	C18:2 n6t	1.4 (0.6)	1.8 (1.0)	0.4 (−0.0, 0.7)	0.07
Linoleic acid	C18:2 n6	66.3 (27.6)	82.5 (37.8)	16.2 (3.7, 28.7)	0.01
Arachidic acid	C20:0	0.9 (0.4)	1.1 (0.6)	0.1 (−0.1, 0.4)	0.18
γ-Linolenic acid	C18:3 n6	0.9 (0.3)	1.0 (0.5)	0.1 (−0.0, 0.3)	0.13
Eicosenoic acid	C20:1 n9	2.7 (1.6)	3.0 (1.3)	0.3 (−0.3. 0.9)	0.32
α-Linolenic acid	C18:3 n3	8.6 (3.1)	10.0 (5.4)	1.4 (−0.4, 3.2)	0.13
Eicosadienoic acid	C20:2 n6	1.6 (0.8)	1.9 (0.9)	0.3 (−0.1, 0.6)	0.09
Behenic acid	C22:0	0.5 (0.2)	0.6 (0.3)	0.1 (−0.1, 0.2)	0.30
Dihomo-g-linolenic	C20:3 n6	2.3 (0.9)	2.6 (1.1)	0.3 (−0.2, 0.8)	0.21
Arachidonic acid	C20:4 n6	2.3 (2.6)	2.6 (1.0)	1.2 (−0.1, 0.8)	0.11
Lignoceric acid	C24:0	0.3 (0.2)	0.4 (0.2)	0.0 (−0.1, 0.1)	0.58
Eicosapentaenoic acid	C20:5 n3	0.5 (0.4)	0.7 (0.7)	0.2 (−0.1, 0.4)	0.25
Nervonic acid	C24:1 n9	0.4 (0.2)	0.4 (0.1)	0.0 (−0.1, 0.1)	0.78
Docosatetraenoic acid	C22:4 n6	0.5 (0.2)	0.6 (0.2)	0.1 (−0.0, 0.2)	0.23
Docosapentaenoic acid	C22:5 n6	0.2 (0.2)	0.2 (0.1)	0.0 (−0.0, 0.1)	0.28
Docosapentaenoic acid	C22:5 n3	1.0 (0.6)	1..2 (0.8)	0.2 (−0.1, 0.6)	0.15
Docosahexaenoic acid	C22:6 n3	1.6 (1.5)	2.0 (1.4)	0.3 (−0.3, 0.9)	0.27
Total fatty acids	-	564 (226)	665 (266)	101 (−16, 218)	0.07

**Table 5:** Plasma fatty acids proportions before and 2 weeks after feeding hindmilk.

Common Name	Plasma Fatty Acids	Before the Start of Hindmilk (%) (*n* = 30)	After Hindmilk (%) (*n* = 27)	*p* Value
Myristic acid	C14:0	1.16 (0.44)	1.20 (0.66)	0.33
Palmitic acid	C16:0	23.56 (1.14)	23.0 (1.15)	0.04
Palmitelaisic acid	C16:1 n7t	0.12 (0.05)	0.13 (0.04)	0.91
Palmitoleic acid	C16:1 n7	1.62 (0.61)	1.07 (0.43)	<0.001
Stearic acid	C18:0	12.24 (0.86)	12.65 (1.46)	0.14
Elaidic acid	C18:1t	0.37 (0.09)	0.39 (0.12)	0.24
Oleic acid	C18:1 n9	22.63 (2.74)	22.67 (3.16)	0.97
Linoelaidic acid	C18:2 n6t	0.19 (0.04)	0.18 (0.05)	0.38
Linoleic acid	C18:2 n6	14.75 (0.89)	16.26 (3.37)	0.002
Arachidic acid	C20:0	0.22 (0.05)	0.22 (0.05)	0.85
γ-Linolenic acid	C18:3 n6	0.24 (0.07)	0.18 (0.07)	<0.001
Eicosenoic acid	C20:1 n9	0.32 (0.05)	0.33 (0.06)	0.17
α-Linolenic acid	C18:3 n3	0.38 (0.17)	0.51 (0.23)	0.007
Eicosadienoic acid	C20:2 n6	0.31 (0.06)	0.31 (0.06)	0.97
Behenic acid	C22:0	0.65 (0.15)	0.57 (0.19)	0.03
Dihomo-g-linolenic	C20:3 n6	2.22 (0.39)	2.01 (0.51)	0.07
Arachidonic acid	C20:4 n6	11.53 (1.36)	10.84 (1.81)	0.10
Lignoceric acid	C24:0	0.54 (0.22)	0.50 (0.20)	0.60
Eicosapentaenoic acid	C20:5 n3	0.49 (0.17)	0.48 (0.22)	0.46
Nervonic acid	C24:1 n9	0.86 (0.38)	0.88 (0.39)	0.36
Docosatetraenoic acid	C22:4 n6	1.22 (0.20)	1.26 (0.27)	0.47
Docosapentaenoic acid	C22:5 n6	0.49 (0.11)	0.45 (0.12)	0.28
Docosapentaenoic acid	C22:5 n3	0.76 (0.19)	0.83 (0.26)	0.29
Docosahexaenoic acid	C22:6 n3	3.09 (0.49)	3.11 (0.64)	0.92

## Data Availability

Deidentified individual participant data will not be made available.

## Supplementary Materials

The following supporting information can be downloaded at: https://www.mdpi.com/article/10.3390/nu15040929/s1. Hindmilk as a rescue therapy in very preterm infants with suboptimal growth velocity.
