# Supplementary material for: Hindmilk as a Rescue Therapy in Very Preterm Infants with Suboptimal Growth Velocity
Source: Nutrients. 2023 Feb 13;15(4):929. doi: 10.3390/nu15040929 (PMC9964728; doi:10.3390/nu15040929)
Supplement: Supplementary file 1 [file nutrients-15-00929-s001.zip › nutrients-2182232-supplementary.pdf]

Supplementary Appendix

Hindmilk as a rescue therapy in very preterm infants with suboptimal growth velocity

Authors: Belal N. Alshaikh[1,2,3], MD, MSc; Jannette Festival[4], RN, IBCLC; Adriana Reyes Loredo[1,5], MD; Kamran Yusuf[3,5], MD, Zainab Towage[1], MD, MD, Tanis R. Fenton[2,3,6], RD, PhD; Christel Wood[7], RN, LC.

Laboratory methods for fatty acid analysis

(OmegaQuant Analytics, SD, USA)

Plasma fatty acid analysis:

For concentration, this is a slight modification (different internal standard) from our published method Hussey et al (12)]. Previously published versions of the plasma percent composition method. (13-15)]

Plasma fatty acid concentration was analyzed by gas chromatography (GC) with flame ionization detection. Plasma was transferred to a screw-cap glass vial which contained tri-tricosanoin (C23:0 TG; NuCheck Prep, Elysian, MN) as an internal standard. The methylating reagent (methanol containing 14% boron trifluoride, toluene, methanol; 35:30:35 v/v/v; Sigma-Aldrich, St. Louis, MO) was added. The vial was briefly vortexed and heated in a hot bath at 100˚C for 45 minutes. After cooling, hexane (EMD Chemicals, USA) and HPLC grade water was added, the tubes were recapped, vortexed and centrifuged help to separate layers. An aliquot of the hexane layer was transferred to a GC vial. GC was carried out using a GC-2010 Gas Chromatograph (Shimadzu Corporation, Columbia, MD) equipped with a SP-2560, 100-m fused silica capillary column (0.25 mm internal diameter, 0.2 um film thickness; Supelco, Bellefonte, PA).

Fatty acids were identified by comparison with a standard mixture of fatty acids (GLC OQ-A, NuCheck Prep, Elysian, MN) which was also used to determine individual fatty acid calibration curves. The C23:0 TG was used to calculate recovery efficiency of the assay and applied to all

fatty acids. The following 24 fatty acids (by class) were identified: saturated (14:0, 16:0, 18:0, 20:0, 22:0 24:0); *cis* monounsaturated (16:1, 18:1, 20:1, 24:1); trans [16:1, 18:1*, 18:2* - see below for more details); *cis* n-6 polyunsaturated (18:2, 18:3, 20:2, 20:3, 20:4, 22:4, 22:5); *cis* n-3 polyunsaturated (18:3, 20:5, 22:5, 22:6). Fatty acid composition was expressed as a percent of total identified fatty acids and concentrations as µg/mL of plasma. The chromatographic conditions used in this study were sufficient to isolate the C16:1*trans* isomers and the C18:2 Δ 9t-12c, 9t-12t, and 9c-12t isomers, which is reported as C18:2n6t. However, each individual C18:1 *trans* molecular species (i.e., C18:1 Δ6 thru Δ13) could not be segregated but appeared as two blended peaks that eluted just before oleic acid. The areas of these two peaks were summed and referred to a C18:1 *trans*.

Breastmilk fatty acid analysis:

Breastmilk fatty acid concentration was analyzed by gas chromatography (GC) with flame ionization detection. Breastmilk was transferred to a screw-cap glass vial which contained 1,2-diheptadecanoyl-sn-glycero-3-phosphocholine as in internal standard (di-C17:0 PL) (Avanti Polar Lipids, USA) and the methylation reagent (methanol containing 14% boron trifluoride, toluene, methanol; 35:30:35 v/v/v; Sigma-Aldrich, St. Louis, MO) was added. The vial was briefly vortexed and heated in a hot bath at 100˚C for 45 minutes. After cooling, hexane (EMD Chemicals, USA) and HPLC grade water were added, the tubes were recapped, vortexed and centrifuged to separate layers. An aliquot of the hexane layer was transferred to a GC vial. GC was carried out using a GC-2010 Gas Chromatograph (Shimadzu Corporation, Columbia, MD) equipped with a SP-2560, 100-m fused silica capillary column (0.25 mm internal diameter, 0.2 um film thickness; Supelco, Bellefonte, PA).

Fatty acids were identified by comparison with a standard mixture of fatty acids (GLC OQ-A, NuCheck Prep, Elysian, MN) which was also used to determine individual fatty acid calibration curves. The di-C17:0 PL was used to calculate recovery efficiency of the assay and applied to all fatty acids. The following 26 fatty acids (by class) were identified: saturated (10:0, 12:0, 14:0, 16:0, 18:0, 20:0, 22:0 24:0); *cis* monounsaturated (16:1, 18:1, 20:1, 24:1); trans [16:1, 18:1*, 18:2* - see below for more details); *cis* n-6 polyunsaturated (18:2, 18:3, 20:2, 20:3, 20:4, 22:4, 22:5); *cis* n-3 polyunsaturated (18:3, 20:5, 22:5, 22:6). Fatty acid composition was expressed as a percent of total identified fatty acids and concentrations as μg/mL of breastmilk.

Note: Concentrations of milk fatty acids were calculated assuming the punches of the saturated filter paper is 10 uL of liquid.

*The chromatographic conditions used in this study were sufficient to isolate the C16:1*trans* isomers and the C18:2 Δ 9t-12c, 9t-12t, and 9c-12t isomers, which is reported as C18:2n6t. However, each individual C18:1 *trans* molecular species (i.e., C18:1 Δ6 thru Δ13) could not be segregated but appeared as two blended peaks that eluted just before oleic acid. The areas of these two peaks were summed and referred to a C18:1 *trans*.
